# Impact of organic and nano selenium on sexual maturation, egg, and offspring quality of European sea bass (*Dicentrarchus labrax*) broodstock

**DOI:** 10.1038/s41598-025-29108-w

**Published:** 2026-01-10

**Authors:** Alaa A. El‑Dahhar, Gomaa A. Khalifa, Samy Y. El‑Zaeem, Mohamed M. Abdel-Rahim, Mona M. Mourad

**Affiliations:** 1 Animal and Fish Production Department, Faculty of Agriculture (Saba Bash), Alexandria University, Alexandria, Egypt; 2 General Authority for Fish Resources Development (GAFRD), Marine Fish Hatchery, Cairo, Egypt; 3 National Institute of Oceanography and Fisheries (NIOF), Cairo, 11562 Egypt

**Keywords:** Selenium, Sexual maturation, Spawning, Egg, Offspring quality, European sea bass, Biochemistry, Biological techniques, Immunology, Physiology

## Abstract

The current research investigated the impact of different types of Selenium fortification in European sea bass (*Dicentrarchus labrax*) broodstock diets on sexual maturation, spawning, egg quality, and offspring quality. The study lasted 85 days and involved 54 broodstock during their maturation period. The broodstock received daily nourishment amounting to 5% of their body weight based on three distinct dietary regimens: a control diet, the basal diet without Selenium supplementation (C), a basal diet supplemented with 0.3 mg of nanoparticle Selenium per kg (N-Se), and a basal diet supplemented with 4 mg of organic Selenium per kg (O-Se). Each dietary regimen was replicated three times. The diets for broodstock were carefully formulated and consisted of squid, dry feed containing 45% protein, and sardines. The broodstock was housed in nine circular fiberglass tanks, each with a capacity of 20 m^3^, accommodating three males and three females per tank. However, each tank had a practical volume of 16 m^3^. The males’ average weight is 1.15 ± 0.05 kg, and measured 41 ± 2 cm in length, while the females’ average weight is 2.0 ± 0.2 kg and measured 52 ± 2 cm. After the feeding treatment, the broodstock was injected with 13 µg/kg of LHRH analog to induce spawning. The water in the tanks was replaced daily with filtered seawater. The tanks were maintained on a 10:30 h light cycle followed by 13:30 h of darkness daily. The water quality was kept within a suitable range for marine fish conditions, including temperature, pH, salinity, and total ammonia nitrogen. The broodstock fed the experimental diets showed significant increases in reproductive performance hormones, including luteinizing hormone (LH), follicle-stimulating hormone (FSH), testosterone (T), and estradiol (E), in both male and female sea bass. Fish-fed diets containing N-Se and O-Se resulted in significantly higher hormone levels (*P* ≤ 0.001) compared to the control diet. Additionally, they exhibited elevated levels of total protein, albumin, globulin, and specific lysozyme activity in their plasma, which was significantly greater than that observed in the control group (*P* < 0.05). In contrast, the control group (Group C) exhibited increased levels of total lipids, cholesterol, and triglycerides. Incorporating Selenium into diets significantly enhanced reproductive performance, egg quality, egg diameter, hatchability, and the size of newly hatched larvae. Fertility rates, egg diameter, oil droplet diameter, and the percentage of fertilized and viable eggs all showed significant improvement when diets were supplemented with nano and organic Selenium compared to the control group. Furthermore, the survival rate and glutathione peroxidase (GPx) levels in larvae from broodstock fed diets enriched with nano and organic Selenium were superior to those observed in the control diet, as indicated in 15 DPH.

## Introduction

The thriving aquaculture industry needs high-quality marine fish larvae. This necessitates accurate oversight and efficient management of the brood fish maturation, spawning, and rearing of offspring phases^1^. Ensuring the nutritional needs of broodstock are vital for successful reproduction, as they profoundly influence larval quality through gamete formation^2^. The mother fish’s nutrition is essential for developing gonads, as it transfers nutrients to the oocytes^3^. Additionally, according to a study by^4^, broodstock preparation is crucial for successful spawning. The survey by^5^ highlighted the significance of Selenium in the diets of broodstock, particularly regarding the structure of selenoproteins, which are essential for antioxidant protection of body tissues and hormone metabolism. Additionally, Se can bolster GPx activity^6,7^. While there has been research on the effects of high-level Selenium supplementation, the impact of Selenium in broodstock nutrition is also crucial. However, the effect of Selenium on metabolism has been investigated in juvenile or larval stages^8,9^. Effective aquaculture should prioritize the production of top-quality larvae, necessitating a thorough comprehension of broodstock nutrition. Within the realm of broodstock nutrition, it remains relatively underexplored and enigmatic. Research indicates that nutrition has a long-lasting physiological impact on the progeny of aquatic animals’ broodstock, as reported by^10,11^, by regulating metabolic processes and shaping the characteristics of the offspring.

Selenium (Se), available in various forms, such as organic and nanoparticle, is essential for healthy and normal aquaculture production. It aids in the synthesis of selenoproteins, which are crucial for generating thyroid hormones and antioxidant substances in tissues while also enhancing immune function^6,12^. Selenocysteine, an amino acid, is essential for forming selenoproteins, which play crucial roles in bodily functions and reproduction^13–15^.. The supplementation of Selenium nanoparticles (Se NPs) enhances the Arabian yellowfin seabream growth^16^. Research shows that various chemical forms of Selenium (Se) affect its bioavailability. Selenium in the form of nanoparticles (Se NPs) has demonstrated increased bioavailability and lower toxicity compared to other forms, as shown in several publications^6,17–19^. Selenium nanoparticles play a crucial role in the normal growth, health, development, and metabolism of aquatic animals^20^.

Investigations into the potential applications of various nanomaterials in medicine and agriculture have been conducted^21,22^. Adequate levels of dietary Se NPs, as reported in several studies, demonstrate that they promote development, protect tissues against oxidative stress, and enhance fish immune responses^17,18,23,24^. Essential selenoenzymes, including glutathione peroxidase (GPx), thioredoxin reductase (TR), and methionine sulfoxide reductase (MSR), possess antioxidant capabilities that defend against oxidative damage by counteracting free radicals and reducing injury to various biological compounds^14^. Various biotic and abiotic stressors can impact fish raised in intensive farming environments. Therefore, incorporating Selenium (Se) as a nutritional antioxidant is essential for maintaining a balanced metabolism in fish raised under these conditions^25^. Impaired antioxidant defense mechanisms can lead to oxidative damage, resulting in oxidation of fish tissues^26^. Because dietary Se significantly impacts growth, reproduction, and offspring survival, previous research has primarily focused on how it affects fish metabolism in broodstock diets^27–30^. E. sea bass is a vital Mediterranean species well-known in global aquaculture for its economic importance, rapid growth, high survival rates in dense stocking conditions, and its ability to withstand a wide variety of environmental fluctuations^31–33^. Several authors recently demonstrated that supplementing Se NPs in aquafeed improves the breedstock reproductive performance for both sexes of Arabian yellowfin seabream^20,34,35^,

The present research investigates the impact of various Selenium sources (organic or nano) on sexual maturation, hormonal levels, reproductive performance, blood biochemistry, spawning, and egg and offspring quality in *European Sea bass (Dicentrarchus labrax) broodstock*.

## Materials and methods

### Ethical statement

All experimental protocols were approved by the Animal Protection and Use Committee of Alexandria University.

All methods of the present work were performed in accordance with the relevant guidelines and regulations.

All methods of the present work are reported in accordance with ARRIVE guidelines.

### The fish husbandry management

A study was conducted on European seabass broodstock from the Marine Governmental Hatchery of the General Authority for Fish Resources Development (GAFRD) in Alexandria, Egypt. A group of 54 broodstock, i.e., 18 brooders per treatment, was introduced into nine circular fiberglass maturation tanks, each capable of holding 20 tons. The tanks had a diameter of 5.05 m and a depth of 1.2 m, containing 16 m³ of seawater, with a water exchange rate set at 200%, equivalent to a flow rate of 22 L/min. Each tank held six fish, maintaining an equal sex ratio (three males and three females). The broodstock are fed daily at 9:30 AM and 1:30 PM with a basal diet (BD) consisting of a mixture of 45% protein dry feed, squid, and sardines, at a feeding rate of 5% of their body weight (BW). Male European seabass broodstock had an average weight of 1.15 ± 0.15 kg and an average length of 41 ± 2 cm. In contrast, female seabass had an average weight of 2.0 ± 0.2 kg, with an average length of 52 ± 2 cm. To stimulate spawning, after the 85-day feeding treatment, both males and females were given an injection of LHRH Analogue (SYNDEL 2595 McCullough Rd, Nanaimo, B.C., Canada at a dosage of 13 mg/kg. This study utilized live European Seabass broodstock, eliminating the need to euthanize the broodstock fish throughout the study period. 50 ppm of MS222 anesthetized the fish before injection and/or blood sampling. Each tank underwent a daily exchange of 100% filtered, well-aerated running Mediterranean seawater, maintaining a natural photoperiod of 10:30 h of light to 13:30 h of darkness from November 9th to February 4th. The water temperature varied between 16 °C and 17 °C, while the pH ranged from 7.7 to 8.0, and salinity levels ranged from 35 to 37 ppt. The concentration of total ammonia nitrogen remained below 0.065 ± 0.006 mg/L.

### Broodstock diets and feeding

The BD contained 15% lipid and 45% crude protein. Broodstock were given feeding treatments for 85 days using three diets: (1) A control diet (C), the BD without Selenium supplementation; (2) The BD supplemented with nano-selenium at 0.3 mg/kg (N-Se), and (3) The BD supplemented with organic Selenium at 4 mg per kilogram (O-Se). A group of 54 broodstock, i.e., 18 brooders per treatment. The prepared diets were thoroughly blended using a laboratory machine. The experimental diets (Table 1), along with their composition and chemical analyses.

**Table 1:** Feed formulation (%) and proximate chemical composition (g kg^− 1^ dry matter) of sea bass broodstock diets supplemented with different sources of selenium.

Ingredients:	C	*N*-Se	O-Se
Wheat flour	10	10	10
Wheat bran	2.6	2.6	2.6
Maize gluten	10	10	10
Soybean meal	20	20	20
Yellow corn	5	5	5
Fish meal	40	40	40
Fish Oil	8	8	8
CMC ^a^	3	3	3
Vit. & Min mix ^b^	1	1	1
Vit. C	0.4	0.4	0.4
TOTAL	100	100	100
Proximate analyses			
Crude Protein	45	45	45
Lipids	15	15	15
Fiber	1.5	1.5	1.5
Ash	8	8	8
NFE ^c^	23.5	23.5	23.5
Total Energy/100g	491.98	491.98	491.98

^1^ BD = Basal Diet; OS = Basal Diet supplemented with organic selenium diet; OS enhanced with 2 gm/kg diets; NS = Basal Diet supplemented with 1 ml Nano selenium Diet (NS)/kg diets.

a. CMC is carboxymethylcellulose.

b. Vitamin & Mineral Mix per Kg Premix: Vitamin A = 4.8 × 106 IU; D3 = 0.8 × 106 IU; Vitamin E = 4 g; Vitamin K = 0.8 g; Vitamin B1 = 0.4 g; Riboflavin = 1.6 g; Vitamin B6 = 0.6 g; Vitamin B12 = 4 mg; Pantothenic acid = 4 g; Nicotinic acid = 8 g; Folic acid = 0.4 g; Biotin = 20 mg; Choline chloride = 200 g; Cu = 4 g; I = 0.4 g; Iron = 12 g; Mn = 22 g; Zn = 22 g; Selenium = 0.4 g.

c. NFE is a nitrogen-free extract.

### Spawning protocol

Following the feeding treatments (85 days), a polyethylene cannula at a diameter of (E.D. 1.25 mm; I.D. 0.86 mm) was used for ovarian biopsies to evaluate the average broodstock egg diameter. The maturation rates of each female’s oocyte were analyzed and categorized based on their size range: late developing (260–400 μm), mid-vitellogenesis (400–600 μm), and late vitellogenesis (600–800 μm). Several hydrated eggs for females were individually recorded (≥ 800 μm) using the methods described by^36^. Females of the present study were found at the mid-vitellogenin stage. Males’ maturity was assessed through stripping of each male and was found to be in the end stage, as described by^37^.

### Blood samples

A 1-mL syringe with 50 µl of heparin was used to collect approximately 0.5 mL of blood samples from each group of broodstock (male and female) while they were in an anesthetized state, with minimal stress, to minimize the impact on the animals. At 4 °C, centrifugation using 750 × g for 10 min was performed on each sample to separate the plasma, which was then stored at − 80 °C for later analysis. MS22 anesthetized the fish before blood sampling, hormonal injection, and other handling procedures.

### Hormonal injection

To stimulate spawning, an injection of 13 µg/kg of LHRH analog was administered to each brooder after the feeding treatment was completed. The LHRH was sourced from SYNDEL at 2595 McCullough Rd, Nanaimo, B.C., Canada. Spawning occurred 60 to 72 h following the injection. The females were sedated using MS22 at a rate of 5 ppm. After sedation, each female was paired with one male from the same group and placed in a 5 m³ spawning tank. Feeding was discontinued during ovulation, and a natural photoperiod of 10:30 light and 13:30 dark was implemented. The tanks were equipped with an egg collector featuring a 300 μm screen mesh.

### Egg samples and incubation

**Fig. 1:**
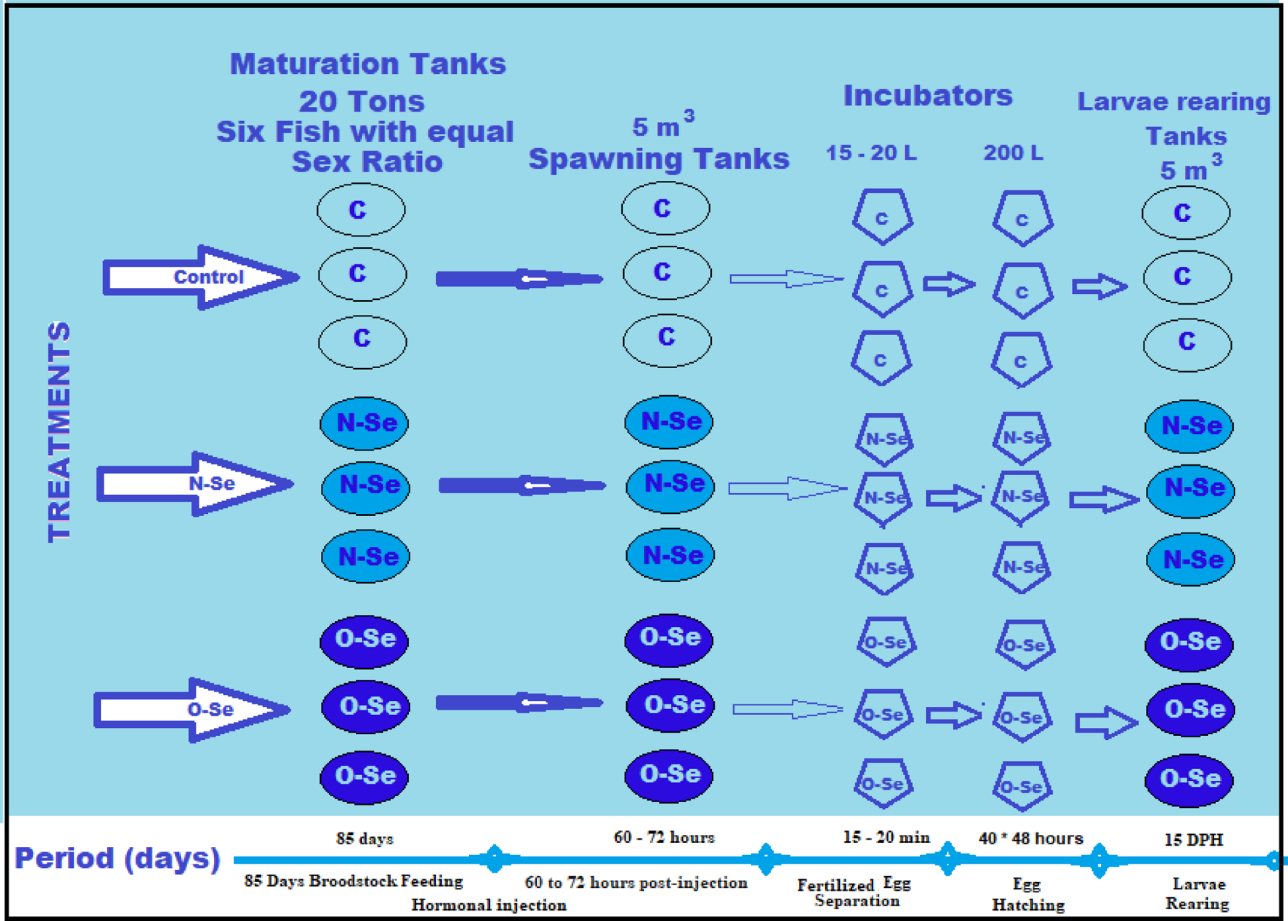
Experimental Periods and Design.

The average diameter of the eggs and oil droplets, as well as the presence of multiple oil droplets within each egg, were examined under a microscope using samples of 200 eggs from each tank. The collected eggs were weighed and placed in a 15- to 20-liter incubator to separate fertilized eggs from unfertilized ones. The fertilized eggs were decontaminated with 0.1 mL of betadine per 30 L before being transferred to nine 200-liter incubation tanks. The density in these tanks was maintained at 5,000 eggs per liter in sterilized filtered seawater. Salinity and temperature were maintained at consistent levels in the spawning tanks. A suitable air stone was used to provide gentle aeration and ensure the eggs were lightly agitated. Water circulation was maintained at a rate of 2 L/min. Each day, artificial aeration in the hatching tank was paused for 1 to 2 min to facilitate the removal of unfertilized or dead eggs from the bottom of the tank. The experimental periods and design of this study are illustrated in Fig. 1.

### Preparing larval rearing tanks

Before receiving the larvae, necessary steps were taken to prepare and maintain the greenhouse. Nine fiberglass tanks in the greenhouse were used, each with a total water volume of 5 m³. The tanks were covered with black polyethylene to control the lighting inside the greenhouse, ensuring sufficient darkness. This experiment involved three treatments, each repeated three times. Tanks containing four cubic meters of water were prepared to receive the newly hatched larvae for each treatment.

### Offspring rearing stage for 15 DPH

The counting of the hatched larvae was conducted in the spawning tanks before their transfer to the larval-rearing tanks, a crucial step in determining the hatching percentage. Samples of 100 ml (5 samples each time) were involved to determine the average number per tank. The larval stocking density was 260,000 larvae per tank (65 larvae per liter), with a 25% daily water exchange. During the three-day post-hatch period, the tanks were kept in complete darkness, as this period is when the larvae are most susceptible to light. A live feed of Rotifer and Artemia was provided during the 15 DPH of the larval rearing process, according to the feeding protocol in the table established by^8^.

### Swim bladder percentage

One hundred larvae samples were randomly collected from each replicate fifteen days post-hatching to evaluate the percentage of swim bladder formation for all treatments under a microscope.

### Reproductive performance and hatchability

The egg diameter (µm).

Egg Maturation (%) = 100 × (Number of mature eggs per Total number of eggs in each sample).

Egg Hydrated (%) = 100 × (Number of hydrated eggs per Total eggs in each sample).

Fecundity (Relative) (10^3^ eggs per kg Body Weight) = Number of spawned eggs per weight of ovulated fish (kg).

Fertility (%) = 100*(Number of fertilized eggs/# eggs per sample).

Non-viable (abnormal; non-floating) Eggs (%) = 100*(# of abnormal eggs/total # of spawned Eggs).

Viable (fertilized; floating) Eggs (%) = 100*(# of normal eggs/total # of spawned Eggs).

Matured (Normal) Eggs (%) = 100*(# of matured developed eggs/total # of eggs sampled before hormonal injection).

Hatchability (%) = 100*(Number of hatched eggs per Number of fertilized eggs in each treatment.

### Reproductive hormones

The levels of FSH hormone in both males and females were measured according to^38^ using ELISA kits with a sensitivity of 0.1 mIU/ml, from Mybioscience, San Diego, CA, USA. Meanwhile, LH hormone was assessed using a quantitative competitive ELISA kit according to^39^ (Mybioscience, San Diego, CA, USA) at a sensitivity of 1.2 mIU/ml. Male testosterone in the serum was measured with a sensitivity of 0.083 ng/ml. In comparison, female estradiol in serum was measured with a sensitivity of 9.71 pg/mL, using a solid-phase enzyme-linked immunosorbent assay (ELISA, DRG Diagnostics GmbH, Germany) based on the principle of competitive binding^40,41^.

### Biochemical parameters in broodstock plasma

The female protein concentration in serum was measured with the biuret method (g/dL)^42^. Albumin (g/dL) using the method described by^43^ was determined by the bromocresol green technique. By subtracting the albumin from the total protein measurement, the Globulin Concentration (g/dL) was calculated. Furthermore, cholesterol concentration (mg/dL) was assessed by hydrolysis and oxidation of the enzyme, as described by^44^. The production estimates lipid concentration (mg/dL) of a pink color as reacted with sulfuric and phosphoric acids, along with vanillin, following the procedure outlined by^45^. Finally, triglyceride levels (mg/dL) were quantified using a colorimetric method that involves a quadruple enzymatic reaction, as described by^46^.

According to^47^, lysozyme activity (µg mg⁻¹ protein) was measured in the serum of both males and females using an agarose gel cell lysis assay. Ly’s plates were prepared at 100 °C with 0.01% agarose dissolved in 0.0067 M PBS (pH 6.3). After the temperature decreased to 60–70 °C, a uniform suspension of 500 mg of Micrococcus lysodeikticus in 5 mL of saline was added to 1 L of agarose and mixed thoroughly. The samples and standard lysozyme solutions were added to separate wells, with 25 µL of each solution poured onto the plates. Afterward, the plates were incubated at 28–30 °C for 15 h, after which the diameters of the clear zones were measured. The dimensions of the clear zones from the samples were compared with the standards to assess the concentration of lysozyme, reported as µg mg^− 1^ protein.

### GPx enzyme activity (U/mg protein)

To measure the activity of the GPx enzyme in larval tissue, we followed the procedure outlined by^48^. This method utilized High-Performance Liquid Chromatography (Reversed-Phase HPLC) from Shimadzu, Hai Zhonglu, Shanghai. We used the assay kit (E-BC-K096-S-Elabscience, Elabscience R Biotechnology Co., Ltd) to evaluate the conversion rate of glutathione (GSH) to oxidized glutathione (GSSG) induced by H2O2 oxidation, thereby indirectly measuring the activity of c-GPx.

The oxidation of NADPH to NADP^+^ was monitored using a Spectrophotometer at 340 nm, and the reduction in absorbance was measured, allowing for the determination of GPx enzyme activity. Using the homogenates of cells or tissues, the extinction coefficient 6220 M-1 cm-1 refers to NADPH at 340 nm. In a mixture of glutathione, NADPH, and glutathione reductase, the activity of c-GPx was evaluated. The addition of hydrogen peroxide as a substrate initiates the enzyme reaction, allowing for the monitoring of the change in absorbance at 340 nm. The activity of GPx in the sample is directly related to the rate of decrease in absorbance.

### Statistical analysis experiment

The data from this study are presented as mean ± standard error (SE) values. The analysis included an examination of reproductive hormones, serum biochemistry, and indicators of reproductive performance, as well as an evaluation of survival rates, growth, and glutathione peroxidase (GSH-Px) levels in European sea bass larvae. A one-way ANOVA was conducted to assess statistical differences between the treatments, and Duncan’s multiple comparison tests were used to evaluate the means, with a significance threshold set at *P* < 0.05. The statistical analysis was performed using SPSS Statistics Standard Version 22 for Windows, developed by IBM (formerly SPSS Inc.) in Chicago, Illinois, as described by^49^.

## Results

### Reproductive hormones in *D. labrax*

**Fig. 2:**
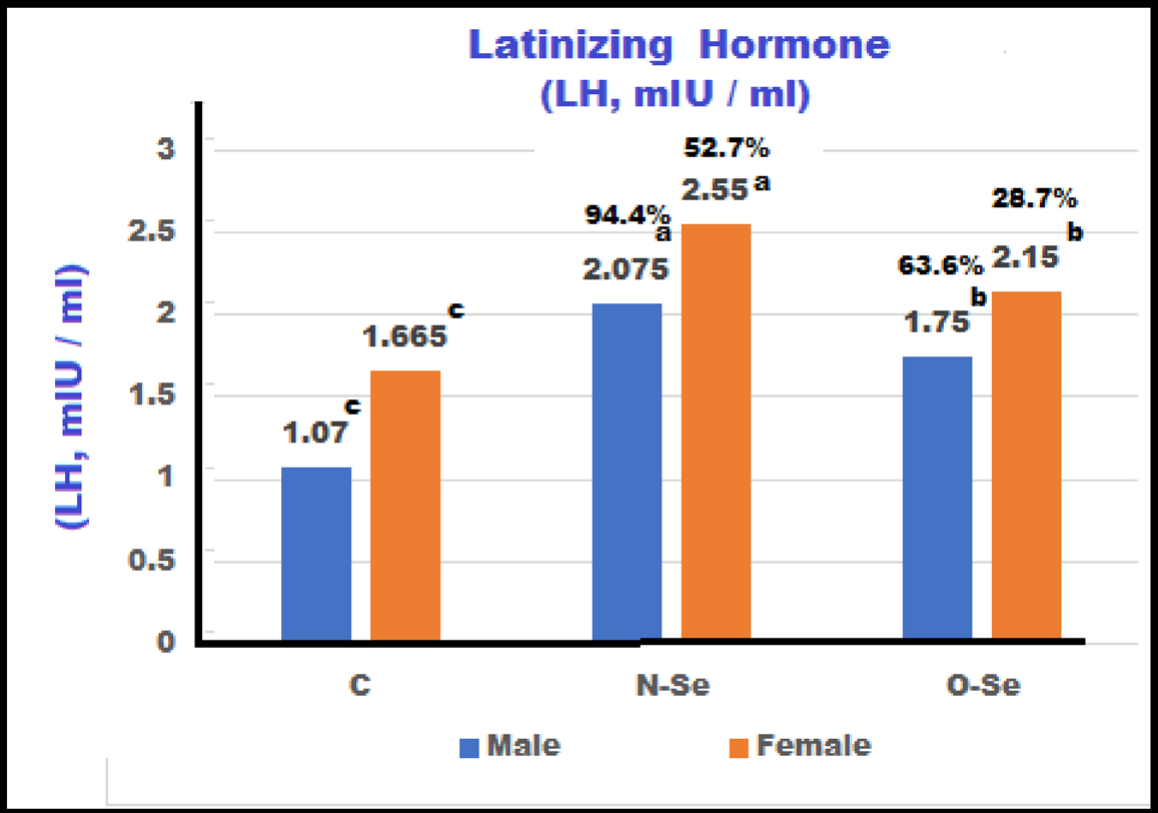
Concentrations of Latinizing Hormones (LH) of male and female European seabass, *Dicentrarchus labrax* broodstock fed with different forms of selenium during maturation (85-day) treatment.

**Fig. 3:**
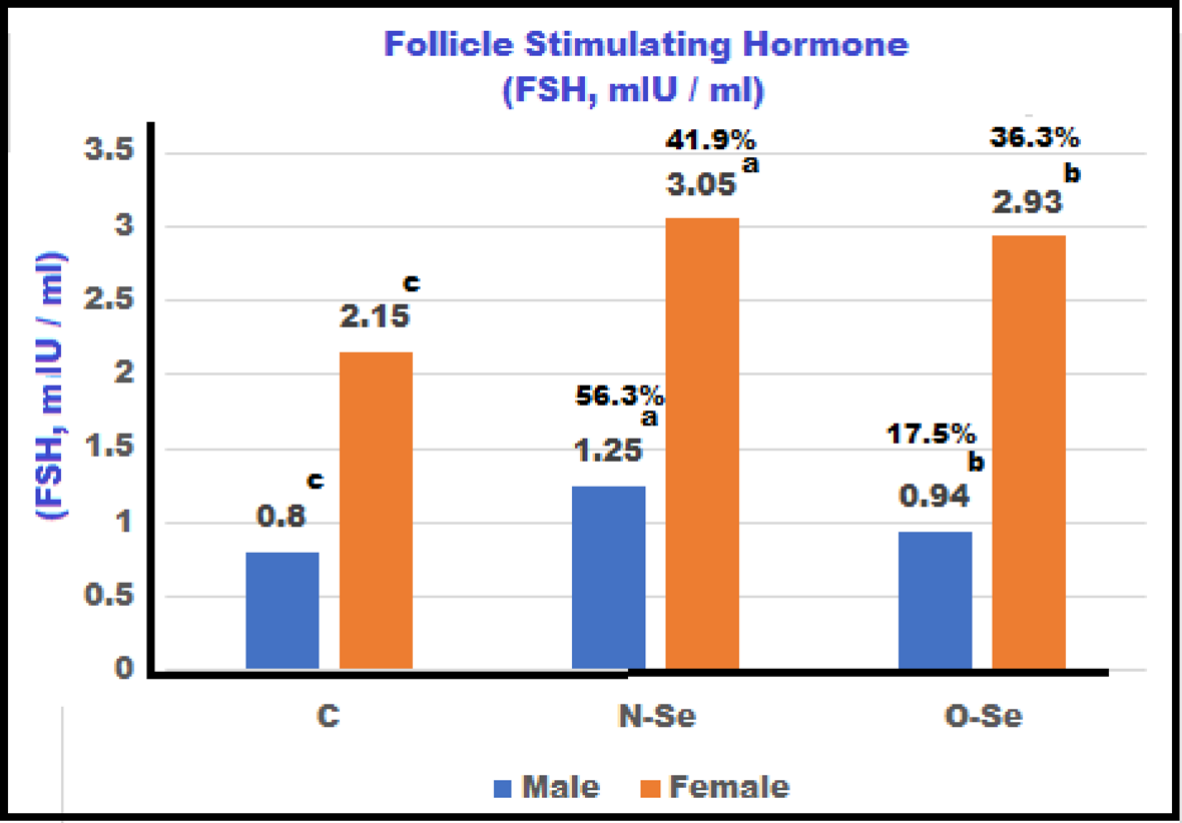
Concentrations of Follicle Stimulating Hormones (FSH) of male and female European seabass, *Dicentrarchus labrax* broodstock fed with different forms of selenium during maturation (85-day) treatment.

**Fig. 4:**
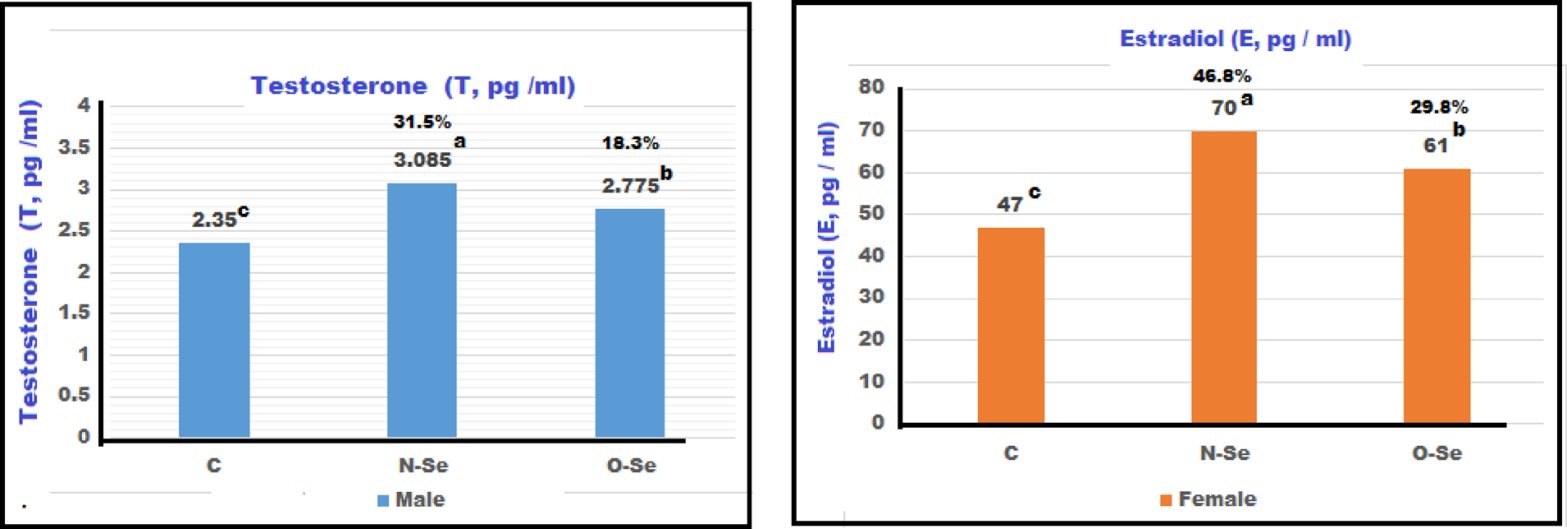
Concentrations of testosterone (T) in males and estradiol (E) in female European seabass (*Dicentrarchus labrax*) broodstock fed different forms of selenium during maturation (85-day) treatment.

The levels of LH, FSH, T, and E in both male and female European seabass broodstock measured after the feeding treatment: control (C), N-Se, and O-Se during the maturation period are illustrated in Figs. 2 and 3, and 4. Males fed the N-Se diet exhibited the highest significant levels of LH, FSH, and T, which were greater than those observed in the control group. Males on the O-Se diet also showed elevated hormone levels compared to the control group, though these were lower than those in the N-Se diet group (*P* ≤ 0.01). Similarly, females consuming the N-Se diet displayed significantly higher levels of LH, FSH, and estradiol compared to those on the O-Se and control diets. Although females on the O-Se diet had substantially higher hormone levels than those in the control group, these were still notably lower than the levels observed in the N-Se diet group (*P* ≤ 0.01). The incremental increases in LH for the N-Se and O-Se diets, compared to the control diet, were 94.4% and 63.6%, respectively. In females, the increases were 52.7% and 28.7% (Fig. 2). For FSH, males experienced increases of 56.3% and 17.5%, while females showed increases of 41.9% and 36.3% (Fig. 3). In terms of testosterone, males had increases of 31.5% and 18.3%. In contrast, female subjects exhibited increases in estradiol levels of 46.8% and 29.8% (Fig. 4). 

### Blood biochemical parameters

The serum parameters (including total protein, albumin, globulin, and lysozyme) of the E. seabass broodstock that were given selenium-enriched diets exhibited notably higher levels (*P* < 0.01) compared to those on the control diet (refer to Table 2). Male seabass on diets enriched with nano- and organic Selenium demonstrated (*P* < 0.01) elevated levels of the plasma total protein (8.0 and 7.3 g/dL), albumin (5.7 and 5.2 g/dL), globulin (2.85 and 2.5 g/dL), and specific lysozyme activity (36.0 and 30.0 µg/mg protein) compared to those of the control group, which reported values of 6.7, 4.2, 1.35, and 8.3, respectively. Furthermore, the data for N-Se significantly exceeded those for O-Se (*P* < 0.01). Similarly, female seabass-fed diets fortified with N-Se and O-Se showed comparable results, with protein levels of 6.5 and 3.7 g/dL, albumin levels of 2.7 and 1.7 g/dL, globulin levels of 3.7 and 2.3 g/dL, and specific lysozyme activity of 14.0 and 12.0 µg/mg protein, respectively. In contrast, the control group exhibited significantly lower values of 2.3, 1.4, 1.1, and 11.5, respectively (*P* < 0.01). In this context, females fed the N-Se diet demonstrated significantly better results compared to the females fed the diet fortified with O-Se (*P* < 0.001).

In contrast, the levels of cholesterol, triglycerides, and lipids in the brood fish fed the selenium-enriched diets (N-Se and O-Se) were significantly lower compared to the control group. Male seabass that consumed diets fortified with nano-selenium and organic Selenium exhibited notably reduced plasma levels of total cholesterol (191.5 mg/dL for N-Se and 287.5 mg/dL for O-Se), triglycerides (84.5 mg/dL for N-Se and 422.0 mg/dL for O-Se), and lipids (641.0 mg/dL for N-Se and 1387.5 mg/dL for O-Se). In contrast, the control group had much higher values, with cholesterol at 307.5 mg/dL, triglycerides at 442.0 mg/dL, and lipids at 1505.5 mg/dL (*P* < 0.01). Similarly, females fed nano- and organic Selenium diets exhibited plasma levels of total cholesterol (87.5 and 208.5 mg/dL), triglycerides (144.5 and 235.0 mg/dL), and lipids (521.0 and 940.0 mg/dL) compared to values of 376.0, 293.0, and 1347.0 mg/dL for these substances of the control group, respectively (*P* < 0.01). In this respect, males and females fed the N-Se diet showed significantly better results compared to the brooders fed the O-Se diet (*P* < 0.001).

**Table 2:** Immune parameters, lipid profile, and lysozyme-specific activity of European Seabass (*Dicentrarchus labrax*) fed the three diets with two forms of selenium and a control for 85 days during the maturation period.

Measured parameters*	Male			*P*-Value	Female			*P*-Value
	C	*N*-Se	O-Se		C	*N*-Se	O-Se	
Total Protein (g/dl)	6.7 ± 0.12^c^	8.0 ± 0.12^a^	7.3 ± 0.15^b^	0.00	2.3 ± 0.12^c^	6.5 ± 0.00^a^	3.7 ± 0.09^b^	0.00
Albumin (g/dl)	4.2 ± 0.12 ^c^	5.7 ± 0.12 ^a^	5.2 ± 0.06 ^b^	0.00	1.4 ± 0.03 ^c^	2.7 ± 0.06 ^a^	1.7 ± 0.09 ^b^	0.00
Globulin (g/dl)	1.35 ± 0.03 ^c^	2.85 ± 0.03 ^a^	2.50 ± 0.00 ^b^	0.00	1.08 ± 0.01 ^c^	3.65 ± 0.03 ^a^	2.25 ± 0.03 ^b^	0.00
Lysozyme (µg/mg protein)	8.30 ± 0.12 ^c^	36.0 ± 1.15 ^a^	30.0 ± 1.15 ^b^	0.00	11.5 ± 0.29 ^b^	14.0 ± 0.58 ^a^	12.0 ± 0.58 ^b^	0.03
Cholesterol (mg/dl)	307.5 ± 1.44 ^a^	191.5 ± 2.02 ^c^	287.5 ± 1.44 ^b^	0.00	376.0 ± 2.31 ^a^	87.5 ± 2.02 ^c^	208.5 ± 1.44 ^b^	0.00
Triglycerides (g/dl)	442.0 ± 1.15^a^	84.5 ± 2.02 ^c^	422.0 ± 1.15 ^b^	0.00	293.0 ± 1.15 ^a^	144.5 ± 1.44 ^c^	235.0 ± 1.15 ^b^	0.00
Lipid (mg/dl)	1505.5 ± 2.60 ^a^	641.0 ± 22.52 ^c^	1387.5 ± 1.44 ^b^	0.00	1347.0 ± 1.73 ^a^	521.0 ± 0.58 ^c^	940.0 ± 1.15 ^b^	0.00

*Data not sharing the same superscript in the same row are significantly different (*P* < 0.05).

### Fecundity, egg quality, and hatching rate

The females fed diets enriched with N-Se and O-Se demonstrated greater relative fecundity, with values of 264.3 × 10^3 and 231.9 × 10^3 per kg of females, respectively, compared to the value of 227.1 × 10^3 per kg of females fed the control group (Table 3). The values of selenium-supplemented diets are 16.3% and 2.1% higher, respectively, than those of the control. The females fed diets fortified with N-Se and O-Se exhibited egg diameters of 1165.33 μm and 1162.33 μm, respectively, which were significantly larger than the 1130.33 μm observed in females fed the Se-in supplemented control diet (*P* < 0.01). The egg diameters were considerably more prominent than those of the control by 3.1% and 2.8%, respectively. Additionally, the oil droplets within the eggs significantly increased by 3.9% and 3.4%, from 334 μm in females fed the control diet to 347.33 μm and 345.66 μm, respectively, for the N-Se and O-Se fortified diets (*P* < 0.05). The viable and fertilized eggs of females fed the Se diets (N-Se and O-Se) are significantly higher than those of females fed a Se-in-supplemented control diet. Viable eggs from the N-Se and O-Se diets exhibited substantially higher values of 366.7 × 10^3 and 296.7 × 10^3 per female, respectively, with fertilization percentages of 84.2% and 77.4%, compared to the control of 258.3 × 10^3 per female, with fertilization percentages of 66.7%. N-Se and O-Se diets are significantly higher in fertilization than the control by 26.3% and 16.1%, respectively.

The non-viable egg counts from the N-Se and O-Se diets were lower, recorded at 68.7 × 10^3 and 86.7 × 10^3 per female, respectively. These data correspond to percentages of 15.81% and 22.62%. In comparison, the control group had 128.3 × 10^3 non-viable eggs per female, with a rate of 33.32%. The Nano and Organic Selenium diets exhibited significant reductions in the non-viable eggs compared to the control, with decreases of 46.4% and 32.4%, respectively (see Table 3).

Mothers fed the N-Se diet get an egg hatchability percentage of 92.69%. It was significantly better than that of the O-Se diet (91.53%) and the control diet (91.0%). The N-Se diet improved hatchability by 1.8% compared to the control and by 1.3% compared to the O-Se diet, which did not differ significantly from the Se-in-supplemented control diet (*P* > 0.05).

Additionally, the newly hatched larvae from mothers fed the Nanoparticle Se diet exhibited significantly higher weights than those fed the Se-in-supplemented control diet (*P* < 0.05). The weights of the newly hatched larvae were 0.484 mg for the N-Se diet and 0.429 mg for the control diet. The larvae from the O-Se diet weighed 0.454 mg, without a significant difference from the N-Se and control diets (*P* > 0.05) (Table 3).

### Larval quality from hatch to 15 DPH

The survival, growth, swim bladder percentage, and Glutathione peroxidase (GPx) activity of 15 DPH larvae from broodstocks fed the three diets with two forms of Selenium and a control for 85 days during the maturation period are shown in Table 4. The larvae from broodfish fed N-Se and O-Se had survival rates of 49.63% and 48.49%, respectively, significantly higher than the control value of 47.02%. The survival rate increased by 5.6% and 3.1% for larvae whose mothers were fed diets fortified with N-Se and O-Se, respectively, compared to those on the control diet.

Larvae from mothers fed on an N-Se supplemented diet exhibited significantly higher swim bladder percentage of 99.33%, compared to 98.66% for O-Se and 97.33% for the control groups. The difference between O-Se and control was significant (*P* < 0.05).

Regarding larval growth, the initial length (IL) at zero DPH was significantly greater for larvae from mothers fed N-Se and O-Se. The measured ILs were 2.23 mm and 2.1 mm, respectively, compared to a control value of 1.9 mm. The final length (FL) at 15 DPH revealed notable differences, with larvae from mothers fed N-Se and O-Se measuring 7.97 mm and 7.67 mm, respectively (*P* < 0.01). Whereas the brooders fed the control diet showed a significantly lower FL of 7.13 mm. The final weight (FW) at 15 DPH for larvae from mothers on N-Se and O-Se diets was also considerably higher, recorded at 2.33 mg and 2.23 mg, respectively, compared to the control value of 1.85 mg (*P* < 0.01). This resulted in an increase of 26.0% for larvae from broodstocks fed N-Se and 20.5% for those fed O-Se, compared to those on the control diet.

Glutathione peroxidase (GPx) activity in newly hatched larvae from broodstocks fed diets enriched with N-Se and O-Se from 0 to 15 DPH exhibited significantly higher values of 12.96 and 11.48 U/mg protein, respectively, compared to the control diet value of 10.51 U/mg protein. The GPx test indicated 22.3% and 9.2% improvements in larvae from broodstocks fed diets enriched with N-Se and O-Se, respectively, compared to those on the Se-in-supplemented control diet (*P* < 0.01).

**Table 3:** Reproductive performance (fecundity and hatching) of European seabass (*Dicentrarchus labrax*) (fed three experimental diets: control diet (C), nano se diet (N-Se), and organic se (O-Se) diet for 85 days.

Measured parameters*	Treatment			*P*-Value
	C	*N*-Se	O-Se	
Female broodstock weight, gm/fish	1703 ± 102	1647 ± 32	1653 ± 29	0.79
Total eggs #/female: 1000	386.7 ± 23.3	435.3 ± 18	383.3 ± 8.8	0.15
Relative Fecundity 10^3^ eggs/kg BW	227.1 ± 23.3	264.3 ± 18	231.9 ± 8.8	0.15
Egg Diameter, µm (after spawning)	1130.33 ± 4.26 ^b^	1165.33 ± 2.6 ^a^	1162.33 ± 2.4 ^a^	0.00
Oil droplet Diameter, µm	334 ± 2.08 ^b^	347.33 ± 1.20 ^a^	345.66 ± 1.45 ^a^	0.02
Total viable eggs #/female: 1000	258.3 ± 20.5 ^b^	366.7 ± 17.9 ^a^	296.7 ± 7.3 ^b^	0.01
Viable (fertilized; floating) Eggs, %	66.68 ± 2.02 ^c^	84.19 ± 1.31 ^a^	77.39 ± 0.18 ^b^	0.00
Total non-viable eggs #/female: 1000	128.3 ± 7.3 ^a^	68.7 ± 5.5 ^b^	86.7 ± 1.7 ^b^	0.00
Non-viable (abnormal; non-floating) Eggs, %	33.32 ± 2.02 ^a^	15.81 ± 1.31 ^c^	22.62 ± 0.18 ^b^	0.00
Hatching, %	91.00 ± 0.29 ^b^	92.69 ± 0.11 ^a^	91.53 ± 0.29 ^b^	0.05
Newly hatched larval weight, mg/larvae	0.429 ± 0.001 ^b^	0.484 ± 0.01 ^a^	0.454 ± 0.001 ^ab^	0.00

*Data not sharing the same superscript in the same row are significantly different (*P* < 0.05).

**Table 4:** Survival, growth, swim bladder percentage, and glutathione peroxidase (GPx) parameters of *Dicentrarchus labrax* larvae (15 days post-hatch) collected from broodstocks fed the three diets with two forms of selenium and a control for 85 days during the maturation period.

Broodstock Treatment*	C	*N*-Se	O-Se	*P*-Value
Survival (0–15 DPH), %	47.02 ± 0.13^c^	49.63 ± 0.34^a^	48.49 ± 0.22^b^	0.00
Swim bladder percentage	98.33 ± 0.67 ^b^	99.33 ± 0.33 ^a^	98.66 ± 0.33 ^ab^	0.05
Initial Length, mm/larvae 0 DPH	1.90 ± 0.058 ^b^	2.23 ± 0.067 ^a^	2.10 ± 0.058 ^ab^	0.02
Final length, mm/larvae 15 DPH	7.13 ± 0.012 ^b^	7.97 ± 0.014 ^a^	7.67 ± 0.012 ^a^	0.01
Final weight, mg/larvae 15 DPH	1.85 ± 0.03 ^b^	2.33 ± 0.03 ^a^	2.23 ± 0.03 ^a^	0.01
GPx (U/mg protein)	10.51 ± 0.110 ^c^	12.86 ± 0.081 ^a^	11.48 ± 0.129 ^b^	0.00

*Data not sharing the same superscript in the same row are significantly different (*P* < 0.05).

## Discussion

The current study suggests that nano- and Organic Selenium have a positive effect on the reproductive hormones of European sea bass broodstock, compared to a control group. Specifically, these supplements influence LH, FSH, T, and E hormone levels in both male and female fish. Brood fish fed diets fortified with N-Se and O-Se showed significant improvements over an 85-day feeding period in comparison to the brooders on the control (Se not fortified diet) (*P* ≤ 0.01). An increase in these reproductive hormones was observed after the 85-day trial when compared to their initial concentrations. The work of^50^ examined the possible influence of sexual steroids on the secretion of gonadotropins in E. Sea bass. The authors noted a complex process regulating gonadotropin secretion. This regulation involves the mechanisms by which steroids are received from the brain, primarily through the gonadotropin-releasing hormone (GnRH) system and the associated feedback mechanisms.

The reproductive processes of fish highlight the critical role of nutrition, as emphasized in research by^51^. Nutrition has a significant impact on the development, reproductive capacity, and survival^52–54^. Additionally^55^,, stated that red tilapia brooders exhibited substantial increases in the relative viscerosomatic index (VSI), gonadosomatic index (GSI), and hepatosomatic index (HSI) when fed diets supplemented with NP-Se. They reported that the highest levels of luteinizing hormone (LH) and estradiol (E2) were observed in female red tilapia fed the NP-Se diet at a concentration of 1 mg/kg. Moreover, fish that received sodium selenite (Na_2_SeO_3_) at a concentration of 1 mg per kg exhibited significantly higher follicle-stimulating hormone (FSH) levels either alone or in combination with NP-Se. Research has shown that the GSI is closely linked to the maturation of the gonads. It peaks during the ripening stage and then declines after females have spawned^56^. Additionally, adding 2–4 mg of nano-selenium per kilogram to a plant-based diet significantly improves normal embryogenesis, larval survival, hatching success, and fertilization rates^20^.

The research findings^57^ enhance understanding of fish reproductive health. The study showed that a diet enriched with Spirulina, which is high in carotenoids, increased estradiol (E_2_) levels in female Pangasius. This enrichment resulted in a greater quantity and weight of their eggs. Estradiol is crucial for vitellogenesis in egg-laying species^58^. Additionally^59^, discovered that fish consuming diets with artemia and Palaemon showed elevated E_2_ levels. These fish also yielded the largest egg diameters and the highest-quality eggs. Moreover, the study on European Sea bass broodstock at the H feeding rate (0.45% of body weight/day) conducted by^60^ produced fewer plasma estradiol and vitellogenin oocytes than the F feeding rate (1.04% of body weight/day). Additionally, the broodstock growth was affected, as evidenced by growth rates in weight and condition factors decreased in group H compared to group F.

The current study investigates immune parameters in European seabass broodstock. It was found that the globulin, albumin, total protein, and specific lysozyme activity levels were significantly higher in male and female fish fed diets enriched with nano- and organic selenium in their plasma compared to the control group (*P* < 0.05). Additionally, the selenium-enriched diets also significantly reduced the broodfish’s cholesterol, triglycerides, and lipid levels after an 85-day feeding treatment compared to those of the control group. In this respect, our results align with previously published data, which indicate that fish fed various levels of Selenium nanoparticles have significantly higher globulin and total serum protein levels compared to those on a basal diet containing 45% crude protein^61^. Our findings support the results reported by^61,62^, which emphasizes that excessive production of reactive oxygen species (ROS) can impair the lipid content of cell membranes and cause damage to RNA. Additionally, elevated ROS levels may lead to lipid peroxidation, which can be measured by the malondialdehyde (MDA)levels.

Our study revealed that organic Selenium (O-Se) decreased overall fats, cholesterol, and triglycerides. Moreover, nano Selenium (N-Se) resulted in even more significant reductions in these markers than the control. Our results are consistent with previously published studies^63^. They found that using nano-selenium particles has effectively decreased cholesterol levels in the blood by impacting cholesterol receptor production to regulate lipid levels and the activity of the 3-hydroxy-3-methylglutaryl coenzyme A (HMG-CoA) enzyme. This suggests that a potent antioxidant in N-Se can enhance the health of the bloodstream. These findings are consistent with those of^64^. Additionally, our findings align with those of^65^. They supported their findings by incorporating vitamin E and nano-selenium particles, or a combination of both, into the feeding of Rainbow trout, which reduced cholesterol levels compared to the control treatment.

Recent reports have shown a growing interest in the use of nanoparticle forms of trace elements in fish diets, particularly for their positive effects on fish health^6^. This interest is supported by the fact that Selenium nanoparticles (Se-NPs) have a larger surface area-to-volume ratio than other Selenium sources, such as sodium selenite, selenomethionine, and methyl selenocysteine. Se-NPs are essential elements that exhibit superior activity and availability within living organisms, along with improved catalytic properties and better particle distribution. Additionally, Selenium is used as a dietary supplement and therapeutic agent in thyroid hormone metabolism, helping to regulate the immune system and enhancing the activity of essential enzymes, particularly antioxidant enzymes. Numerous researchers have been investigating the various effects of Selenium nanoparticles (Se-NPs) on fish, focusing on their impacts on growth, blood parameters, biochemical properties, immune responses, antioxidant activity, and potential toxicity^66–69^. Higher blood levels indicate good health, disease resistance, and lower stress. Likewise, incorporating Selenium supplements into the diet is essential for supporting fish health. These supplements enhance the fish’s ability to combat free radicals, protect cells and tissues, and improve metabolic efficiency^16,70–72^.

In the current study, nano- and organic Selenium in the broodstock diet improved their fertility rates by 26.3% and 16.1% respectively, and relative fecundity by 16.3% and 2.1%, respectively, compared to the control. This dietary supplementation also resulted in larger egg diameters, increased oil droplet diameters, higher fertilized and viable eggs, decreased non-viable eggs, and a higher hatching % than the control. Fecundity, a vital indicator of a broodfish’s reproductive performance, is influenced by the fish’s nutritional status, as found by^73,74^. In a study conducted by^20^, it was found that a positive correlation exists between Selenium (Se) levels in a diet based on plant protein and larval survival, hatching rates, and fertilization rates. Specifically, they reported increases in fertilization success of 24.38%, 51.31%, and 67.95% with the addition of nano-Se supplementation at 1, 2, and 4 mg/kg, respectively, in the plant protein-based diet. These findings align with our results, which showed increases of 26.3% and 16.1% when N-Se and O-Se were added to the basal diet.

The present study demonstrates a positive effect of N-Se and O-Se fortification in the diet on larval survival rates, initial length, and swim bladder percentage at 15 days post-hatching (DPH). These results also demonstrate that the broodfish diet supplemented with N-Se and O-Se can effectively enhance the egg and offspring quality by providing an essential Se pool within the eggs during the embryonic period. Our results align with those reported by^75^. They found that total Se levels in the larval body were influenced by their mother’s Se nutrition and direct Se feeding compared with the control. As reported by ^**24**^, the results showed a significantly higher weight gain and specific growth rate in fish fed diets with lysolecithin than in those on the control diet. The quality of larvae and eggs is vital in aquaculture, as it supplies high-quality eggs, necessary for the proper growth of the cultured species^51,76^. The nutritional history of mothers during oogenesis affects the biochemical composition of fish eggs, which is essential for normal embryonic and larval development^77^. They identified the optimal spawning parameters for *Paralichthys olivaceus* by adding 10% haddock visceral oil, resulting in higher concentrations of HUFA n-3 in the eggs and an increased lipid percentage of 23.46%.

The current study revealed that females fed N-Se and O-Se diets experienced a negligible increase in egg production and relative fecundity compared to those in the control group. These findings align with the results of^75^. They found no notable differences in the fecundity of rainbow trout fed a diet based on plant protein supplemented with Selenium. The study of^78^, also found similar results when working with rainbow trout. They found significant egg variation in zebrafish fed diets containing 0.09 or 0.65 mg Se/kg. The research conducted by^34^ observed an increase in the number of eggs spawned daily by female fish as the total Selenium content increased. This finding suggests a positive effect on the reproductive success of Arabian yellowfin sea bream fed a diet high in plant protein enriched with 4 mg of NP-Se/kg by enhancing egg production^20^.

The weight of newly hatched larvae in the current study increased in groups supplemented with N-Se and O-Se fortification compared to the control group. When assessing progeny quality, factors such as egg viability, hatching percentage, and survival rates are crucial, as highlighted by^51^. They also pointed out that certain specific nutrients, such as vitamin E, C, and carotenoids, are necessary for fish reproduction^79,80^. In salmon, vitamin C has been identified as crucial for reproduction, supported by findings from^81,82^. During the maturation and spawning stages, the developing gonads require numerous nutrients to facilitate egg production and the spawning activities of fish brooders^83^. Consequently, as stated by several research studies^84,85^, enhancing diets with proteins, fats, vitamins, and inorganic materials ensures that fish growth and survival remain unaffected.

Our study investigated the impact of feeding parent fish diets containing nano-selenium and organic Selenium on the GPx activity in their offspring from hatching to 15 days post-hatch. The findings indicate that larvae from mothers fed diets enriched with N-Se and O-Se exhibited 22.3% and 9.2% improvements, respectively, compared to those fed the control diet, with significantly higher GPx activity (*P* < 0.01). Our results support the findings of^34^. They reported that males of sea bream (*Acanthopagrus arabicus*) fed diets with various levels of nano-selenium (0 to 4 mg per kg) in a plant protein-based diet resulted in the highest semen GPx activity in those receiving 4 mg of nano-selenium/kg. The research conducted by^86^ arrived at similar results for GPx activity in swim-up fry-fed both diets enriched with Selenium in different forms, the highest levels found in the selenomethionine treatment. Furthermore^8^, found that the larvae of E. sea bass fed diets supplemented with Selenium in nano or organic forms, a 60-day treatment, demonstrated significantly higher GPx activity (*P* < 0.05) than those fed a control diet without Selenium supplementation. On the other hand, the finding of^16^ which indicates that dietary Se NPs for Arabian yellowfin seabream did not significantly affect the superoxide dismutase (SOD) activity and MDA concentration. Still, it enhanced the catalase (CAT) and GPx activity in groups supplemented with Se NPs at 0.5 and 1 mg/Kg. They also discovered that GPx activity levels were not significantly affected by adding nano-Se at doses of 0.5, 1, or 2 mg/Kg for 60 days. However, they observed a low level of MDA in the Se-2 group.

Selenium nanoparticles play a crucial role in producing antioxidative enzymes, thereby enhancing the overall antioxidative capacity. Research by^12^ demonstrated the improved activities of MDA, GPx, CAT, and SOD due to the use of nano-Se. Additionally, feeding fish with nano-Se compared to the diet without Se fortification showed increased levels of lysozyme and IgM. These findings align with previous results demonstrating that nano-Se supplementation can enhance antioxidant measurements in common carp and Nile tilapia^87,88^. Additionally^89^, found that European sea bass exhibited decreased oxidative stress and improved oxidative enzyme activity with a nanoparticle selenium-fortified diet. Integrating Selenium nanoparticles into the diet enhances cellular defense against oxidative stress and strengthens antioxidant protection in fish when they encounter disease-causing pathogens^90^. Moreover^91^, research revealed that Nile tilapia fed a nano-Se diet at 0.4 or 0.8 mg/kg showed reduced MDA activity and improved the performance of antioxidant enzymes.

Selenium (Se) provides antioxidant protection through selenium-dependent GPx production^92^. The study conducted by^93^ showed that a diet deficient in Selenium typically reduces the antioxidant mechanism by the GPx activity in aquatic organisms, with the liver GPx activity being a dependable indicator of insufficient nutritional Selenium. However^86^, found that hydroxyl selenomethionine in parental nutrition enhanced the gene expression of the SelP, GPx, and methionine Sulfoxide Reductase B2 antioxidative proteins, which increased the GPx activity in the offspring. Moreover^94^, found that GPx activity in semen increased with dietary Se-N, MDA levels, and the motility of sperm lifespan, suggesting that GPx may enhance sperm’s ability to withstand ROS. In the same direction^95^ stated that addressing lipid peroxidation in common carp sperm occurred through the activity of GPx as the primary antioxidant enzyme. The ability of antioxidants includes both enzymatic and non-enzymatic types, which are utilized to evaluate sperm quality and gauge fertility in teleost fish. The seminal plasma levels differ across species, indicating a physiological trait specific to each species^96^.

## Conclusion

The present study on broodstock maturation indicates that incorporating the antioxidant selenium in both nanoparticulate and organic forms in the broodstock diet can significantly enhance reproductive performance in both male and female sea bass. The diets supplemented with selenium (N-Se and O-Se) resulted in significantly higher levels of reproductive hormones, including luteinizing hormone (LH), follicle-stimulating hormone (FSH), testosterone (T), and estradiol (E), than the control group. Additionally, the selenium-enriched diets significantly increased the plasma total protein, albumin, globulin, and specific lysozyme activity levels compared to those observed in the control group. In contrast, the selenium-enriched diets significantly decreased the total plasma lipids, triglycerides, and cholesterol compared to the control. Overall, incorporating Selenium into the diets markedly improved reproductive performance, egg quality, egg diameter, hatchability, and the size of newly hatched larvae. It also enhanced the growth and survival rates of European seabass larvae. Furthermore, this approach enhanced antioxidant effectiveness, increased larval glutathione peroxidase (GPx) activity, and promoted anti-inflammatory effects. Based on these findings, we recommend utilizing a nanoparticulate diet of 0.3 mg/kg and/or an organic Selenium diet of 4 mg/kg to enrich the broodstock and larval diets of European seabass, thereby supporting their production.

## Data Availability

The datasets used and/or analyzed during the current study are available from the corresponding author on reasonable request.
